# Hypoxia-induced miR-191-C/EBPβ signaling regulates cell proliferation and apoptosis of fibroblast-like synoviocytes from patients with rheumatoid arthritis

**DOI:** 10.1186/s13075-019-1861-7

**Published:** 2019-03-20

**Authors:** Shanshan Yu, Ying Lu, Ming Zong, Qi Tan, Lieying Fan

**Affiliations:** 1 Department of Clinical Laboratory, Shanghai East Hospital, Tongji University School of Medicine, 150 Ji Mo Road, Shanghai, 200120 People’s Republic of China; 2 Research Center for Translational Medicine, Shanghai East Hospital, Tongji University School of Medicine, 150 Ji Mo Road, Shanghai, 200120 People’s Republic of China

**Keywords:** miR-191, CCAAT/enhancer binding protein β, Hypoxia, Rheumatoid arthritis, Fibroblast-like synoviocytes

## Abstract

**Background:**

Hypoxia plays an important role in the proliferation of rheumatoid arthritis fibroblast-like synoviocytes (RA-FLS), leading to pathology of RA. This study was conducted to evaluate hypoxia-induced microRNAs (hypoxamiR) in RA-FLS and its role in the function of RA-FLS.

**Methods:**

RA-FLS were cultured under normoxia (21% O_2_) or hypoxia (3% O_2_) condition, followed by a microRNA (miRNA) array analysis. The upregulation of miR-191 by hypoxia was confirmed in RA-FLS and FLS from osteoarthritis (OA) patients by quantitative real-time polymerase chain reaction (RT-PCR). Transfection of miR-191 mimic and inhibitor was used to investigate the function of miR-191 in RA-FLS. The functional targets of miR-191 were predicted by bioinfomatics and then validated by reporter gene assay.

**Results:**

A subset of miRNAs was identified to be induced by hypoxia including miR-191. The upregulation of miR-191 was found to be specific in hypoxic RA-FLS, compared to hypoxic OA-FLS. We observed that miR-191 in RA-FLS increased cellular proliferation via promoting G_1_/S transition of the cell cycle and suppressed cell apoptosis induced by cell starvation. Bioinformatical analysis and experimental assays identified CCAAT/enhancer binding protein β (C/EBPβ) as a target gene of miR-191 in RA-FLS. Enforced expression of C/EBPβ rescued the cellular phenotypes induced by miR-191. In addition, an inverse correlation between the C/EBPβ level and hypoxia stimulation was found in RA-FLS, and overexpression of C/EBPβ could partly rescue the hypoxia-induced cell proliferation.

**Conclusion:**

We demonstrated the miR-191-C/EBPβ signaling pathway mediating the hypoxia-induced cell proliferation in RA.

## Background

Rheumatoid arthritis (RA) is a systemic autoimmune disease, which is characterized by chronic joint inflammation and synovial hyperplasia. It is estimated that there are approximately 1% of population being affected by this disease worldwide [1]. Rheumatoid arthritis fibroblast-like synoviocytes (FLS), as the main stromal cell population in the joint synovium, proliferate and invade to destroy the adjacent cartilage [2, 3]. The mechanisms regulating the aberrant growth of RA-FLS remain unclear, although quite a few studies have been reported from literature indicating the hypoxia environment in the joint may be the main reason causing inflammation and hyperplasia in RA [4–6].

In comparison with RA, osteoarthritis (OA) is a degenerative condition that is the result of increased wear and tear on joints [7, 8]. Since the inflammatory symptoms, joint damage and fibroblast-like synoviocytes proliferation are all more severe in RA than that in OA, OA has been frequently used as control to study the pathobiology of RA.

Reduced oxygen and induced inflammation in the synovium of arthritis have been well demonstrated as the key role in the progression of RA [9]. In the inflamed joint, the level of hypoxia is inversely correlated with the levels of vascularity, oxidative damage, and synovial inflammation [10, 11]. Hypoxia induces angiogenesis and promotes cell growth by regulating the expression of the key genes, such as hypoxia-inducible factors (HIFs) and vascular endothelial growth factor (VEGF) [12]. Our previous study also demonstrated that hypoxia promoted cellular proliferation of RA-FLS and angiogenesis in RA though upregulating glucose-6-phosphate isomerase (G6PI) [13].

In addition, microRNAs (miRNAs) have also been found to express abnormally under hypoxia condition, which is closely related with cell types [14–16]. miRNAs are a class of small non-coding RNA molecules regulating the stability or translational efficiency of targeted messenger RNAs, participating in a wide range of biological processes such as cellular proliferation, differentiation, and apoptosis [17]. miR-155 and miR-146a are the most well-studied miRNAs in RA, significantly upregulated in RA-FLS, and take roles in RA progression and development [18, 19]. However, the miRNA expression profile and the potential function of hypoxia-regulated miRNAs in RA-FLS have not been systematically studied yet.

In this study, we performed a miRNA screen analysis in the hypoxia-exposed RA-FLS and identified miR-191 induced by hypoxia condition. Overexpression of miR-191 promoted cellular proliferation and protected RA-FLS from apoptosis. CCAAT/enhancer binding protein β (C/EBPβ) was demonstrated to be a target gene of miR-191, mediating the regulatory function of miR-191 in RA-FLS.

## Methods

### Specimen collection and HE staining

Synovial tissues from RA (*n* = 5) and OA (*n* = 5) patients were fixed in 10% neutral buffered formalin and embedded in paraffin and then cut into 5-um-thick sections, and stained with hematoxylin and eosin (HE), following the manufacturer’s introduction. All patients fulfilled the diagnosis of the American College of Rheumatology (ACR) for RA and OA. Informed consent was obtained from each of the enrolled patients, and the study protocol was approved by the Ethics Committee of Shanghai East Hospital (2016-df-011). Prior to tissue collection, signed informed consent was obtained from each patient. This study was conducted in accordance with the guidelines of the Declaration of Helsinki.

### Isolation and culture of fibroblast-like synoviocytes

Synovial tissues from RA or OA patients were immediately placed in RPMI 1640 medium (Life Technologies, Carlsbad, CA, USA) and processed within 4 h. The tissues were minced and evenly spread on the bottom of cell culture flasks in RPMI 1640 medium at 37 °C for 6 h. Next, the tissues were incubated with RPMI 1640 medium supplemented with 10% fetal bovine serum (FBS) and penicillin (100 U/ml) and streptomycin (100 μg/ml) at 37 °C in a humidified 5% CO_2_ atmosphere. Non-adherent tissue pieces were carefully removed by replacing the medium every 3 to 5 days and passaged when the primary synoviocytes reached 70–80% confluence. FLS grown over four to eight passages and were used for further analysis. FLS were cultured under normoxia condition and/or 3% O_2_ hypoxia condition in BioSpherix oxygen control system.

### RNA quantification and real-time PCR analysis

Total RNA of FLS was extracted using TRIzol™ (Invitrogen, Carlsbad, CA, USA), after culture under the conditions of normoxia or 3% O_2_ hypoxia for 24 h. An M&G miRNA Reverse Transcription Kit (miRGenes, Shanghai, China) was used to prepare the first strand cDNA of miRNAs following the manufacturer’s instruction. One hundred nanograms of purified total RNA from each sample was used for miRNA measurement. After reverse transcription, the cDNA was diluted 1:1,000 for quantitative real-time polymerase chain reaction (RT-PCR). The miRNA profiling analysis was performed with the quantitative RT-PCR-based miRNA panel which contains 365 miRNAs and 2 reference small RNAs (5 s ribosomal RNA and u6) on an ABI PRISM 7900 (Applied Biosystems, Foster City, CA, USA). Forward primer sequences for RT-PCR of miRNAs were miR-191: 5′ gaatcccaaaagcagctg 3′; miR-297: 5′ atgtgtgcatgtgcatg 3′; miR-499b-3p: 5′ aacaucacugcaagucu 3′; miR-770: 5′ uccaguaccacgugucag 3′; miR-936: 5′ acaguagagggaggaaucg 3′. For C/EBPβ mRNA detection, total RNA was reverse transcribed to complementary DNA (TaKaRa, Dalian, China) according to the manufacturer’s instructions. Gene expression was analyzed by relative quantification using Premix Ex Taq SYBR Green PCR (TaKaRa) on an ABI 7500 Real Time PCR System (Applied Biosystems). The sequences of primers were used as follows: C/EBPβ, forward: 5′-TTCAAGCAGCTGCCCGAGCC-3′, reverse: 5′-GCCAAGTGCCCCAGTGCCAA-3′; and GAPDH, forward 5′- TGACTTCAACAGCGACACCCA-3′, reverse 5′-CACCCTGTTGCTGTAGCCAAA-3′. GAPDH served as the internal control. All RT-PCR reactions were analyzed using Relative Expression Software Tool (REST®) 2009 based on 2^−ΔΔCt^ method.

### Transfection of siRNA and plasmid

Mimic-miR-191 and its control (control-mimic), and antisense oligo nucleotide targeting miR-191 (inhibitor-miR-191) and its control (inhibitor-control) were synthesized by ribobio Co. (Guangzhou, China). The target sequences for C/EBPβ siRNA (si-C/EBPβ)-1 (5′-CCG TGG TGT TAT TTA AAG A-3′), si-C/EBPβ-2 (5′-CCC TGA GTA ATC GCT TAA A-3′), and si-negative control (si-NC) (5′-TTC TCC GAA CGT GTC ACG T-3′) were synthesized by Genepharm (Shanghai, China). The plasmids of pEnter and pEnter-C/EBPβ were synthesized by Weizhen (Shandong, china). The transfection of siRNAs and plasmids was performed using Lipofectamine® 2000 (Invitrogen, Carlsbad, CA, USA) following the manufacturer’s protocol. Final concentration of 30 nM of small RNA oligos was used for all in vitro assays.

### Cell proliferation assay

RA-FLS that had been transiently transfected with small RNA oligos (30 nM) or plasmids (2 μg/ml) for 24 h were seeded on 4 × 10 ^3^ cells/well in 96-well plates and incubated under conditions of normoxia or 3% O_2_ of hypoxia. At the end of each time period (as indicated in the figures or figure legends) from transfected cells being seeded, cell proliferation was determined by using the Cell Counting Kit-8 (CCK8) (Donjindo, Japan) according to the manufacturer’s instructions. Briefly, 20 μl CCK8 was added to each well containing 200 μl medium and then incubated at 37 °C for 2–4 h. The absorbance was read at 450 nm on a spectrophotometric plate reader (Bio-Rad, Hercules, CA, USA). Each assay was performed in quintuplicate, and all tests were repeated three times.

### Cell cycle and cell apoptosis analysis

RA-FLS were transfected with the indicated oligos or plasmids in six-well plates. For cell cycle analysis, the cells were collected after 48 h of transfection. The cells were washed with PBS and then fixed with 70% ice-cold ethanol overnight at − 20 °C, washed with PBS, resuspended with 400 μl PBS, and then incubated with 100 μg/ml RNaseA (Kaiji, China) for 30 min at 37 °C and with 50 μg/ml propidium iodide (PI) (Kaiji, China) for another 30 min at 4 °C. After incubation, the cells were subjected to DNA content analysis using BD FACS Calibur cytometry and the results were analyzed with the ModFitLT software. For apoptosis analysis, the complete medium was changed to RPMI 1640 medium supplemented with 1% FBS to induce cell apoptosis, after transfection for 24 h. Cell apoptosis was evaluated by Annexin V-FITC and PI (BD Biosciences, 556547) staining according to the manufacturer’s protocol, followed by flow cytometry analysis. Briefly, cells were collected and washed with ice-cold PBS and resuspended in 100 μl binding buffer. Then, 5 μl of Annexin V-FITC and 5 μl of PI were added to the cells, incubated for 15 min at room temperature in the dark, and an additional 400 μl of binding buffer was added to the reaction prior to analysis. The results were analyzed with the Summit v4.3 software.

### Western blot analysis

Cells were lysed in lysis buffer and centrifuged at 12,000 rpm for 10 min. The protein samples in the supernatant were immediately collected, and the concentration was measured using the Bradford method (Bio-Rad, Hercules, CA, USA). Equal amounts of protein were separated by 10% SDS-PAGE and then transferred onto PVDF (polyvinylidene fluoride) membranes (Amersham Pharmacia Biotech, Uppsala, Sweden). After blocking with 5% nonfat dry milk in PBS containing 0.1% Tween-20 for 2 h at room temperature, the membranes were incubated at 4 °C overnight with primary antibodies (1:1000) against cyclinD1 (#2978, cell signaling), CEBP/β(#3087, cell signaling), and β-actin (sc-47778, Santa Cruz) and subsequently incubated with secondary horseradish peroxidase antibody. The immunoreactive proteins were visualized using an enhanced chemiluminescent reagent (Millipore Corporation, USA).

### Transwell migration assay

RA-FLS that had been transiently transfected with inhibitor-control or inhibitor-miR-191 were plated in transwell invasion chambers (Corning) on membranes precoated with Matrigel (Corning) containing RPMI 1640 medium supplemented with 1% FBS, and RPMI 1640 medium supplemented with 5% FBS in the lower wells. After a 24-h incubation, Matrigel were removed with a cotton swab, and the cells were fixed and stained with 0.1% crystal violet solution and assessed by two observers in a blinded manner.

### Luciferase reporter assay

293T cells were seeded on 24-well plates at a density of 1 × 10^5^ cells. Then, cells were transfected with 1.0 μg WT or pMIR-REPORT plasmid (Obio, Shanghai, china) and 100 ng Renilla plasmid, together with mimic-miR-191/mimic-ctrl (30 nM). After a 24-h transfection, reporter assays were performed using the Dual Luciferase kit (Promega, Madison, WI, USA) by AutoLumat.

### Statistical analysis

SPSS 20.0 program package (SPSS Inc., Chicago, IL, USA) was used for all statistical analyses. Data are presented as mean ± SEMs. The standard two-tailed Student’s *t* test was used for analysis, in which *p* < 0.05 was considered statistically significant.

## Results

### miRNA expression screening in hypoxic RA-FLS

**Fig. 1:**
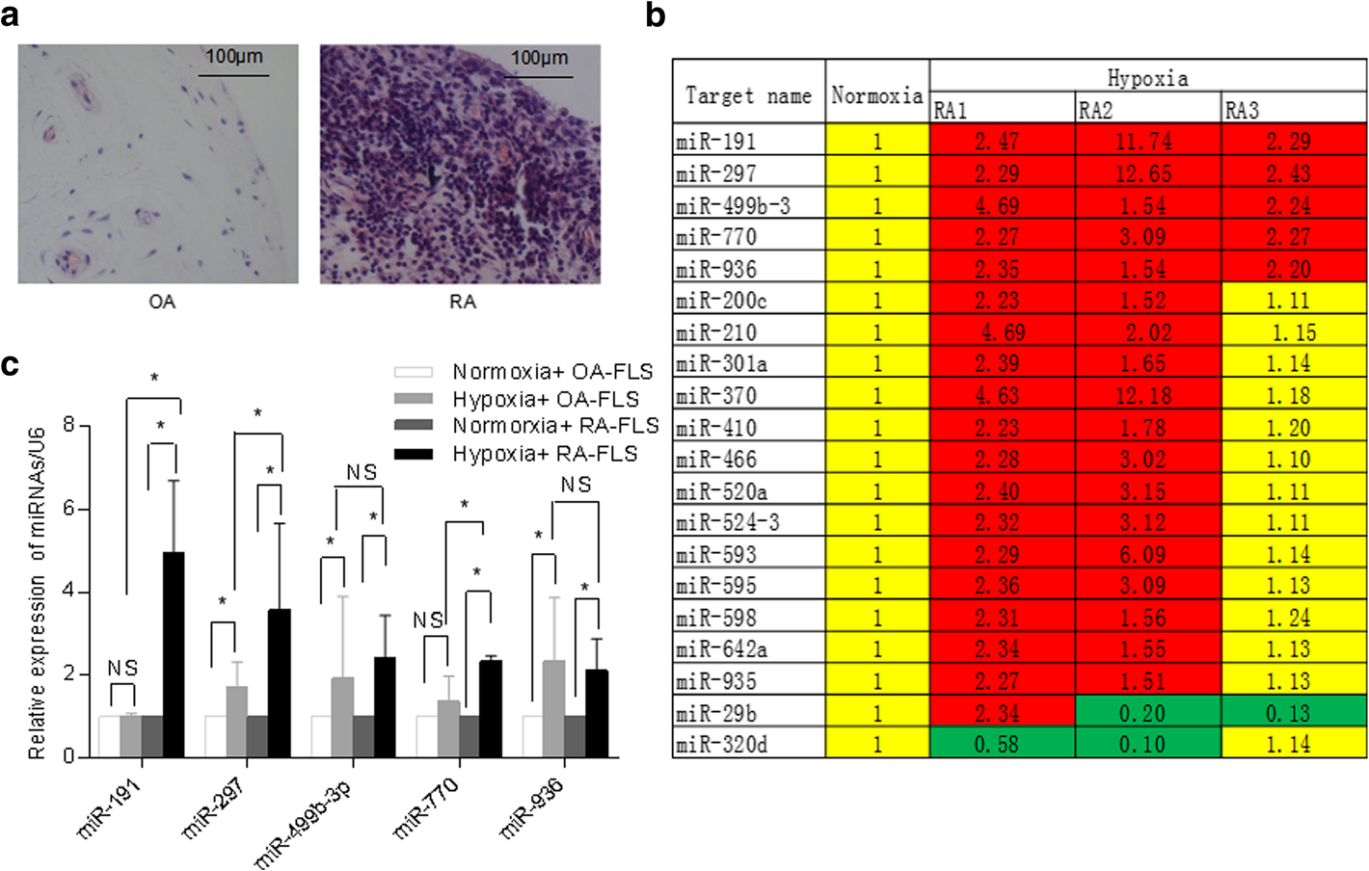
Upregulation of miR-191 in hypoxic RA-FLS. **a** HE staining of synovial tissue samples from RA and OA patients. **b** miRNA profiling analysis for RA-FLS, cultured under normal or hypoxia conditions for 24 h, identified a subset of dysregulated miRNAs. Data was represented as fold change of miRNAs expression in hypoxic RA-FLS, compared to normoxic RA-FLS. **c** Further validation of the miR-191, miR-297, miR-499b-3p, miR-770, and miR-936 expression in three RA-FLS cells and three OA-FLS cultured under hypoxia condition for 24 h, compared to normoxia condition by quantitative RT-PCR. Data represent results from three independent experiments, shown as means ± SEMs (*n* = 3, *p < 0.05 by *t* test)

The synovial tissue samples from patients with RA (*n* = 5) and OA (*n* = 5) were collected, paraffin-embedded, and sectioned, followed by HE staining analysis. As shown in Fig. 1a, RA patients showed inflammation in the lining of the joint and synovial hyperplasia, compared to OA patients. In order to determine the potential role of hypoxia-induced microRNAs (hypoxamiR) in RA-FLS, primary RA-FLS from three patients were isolated and cultured under normal or 3% O_2_ of hypoxia for 24 h. Quantitative RT-PCR-based miRNA profiling analysis were performed and compared between normal and hypoxia conditions. Figure 1b showed that, out of a total of 365 miRNAs, 5 showed significant upregulation more than 1.5 fold change in all 3 RA-FLS cell lines by hypoxic culture including miR-191, miR-297, miR-499b-3p, miR-770, and miR-936, while only 2 of the 365 miRNAs showed downregulation in 2 of the 3 samples including miR-29b and miR-320d. Furthermore, the expression of the five upregulated miRNAs was compared in three RA-FLS and three OA-FLS cell lines cultured under normal or hypoxia condition. The expression of miR-191 and miR-770 was found to be specifically upregulated in RA-FLS, whereas other three miRNAs (miR-297, miR-499b-3p, and miR-936) showed upregulation by hypoxia treatment in both RA-FLS and OA-FLS (Fig. 1c).

### miR-191 promoted the cellular proliferation of RA-FLS

**Fig. 2:**
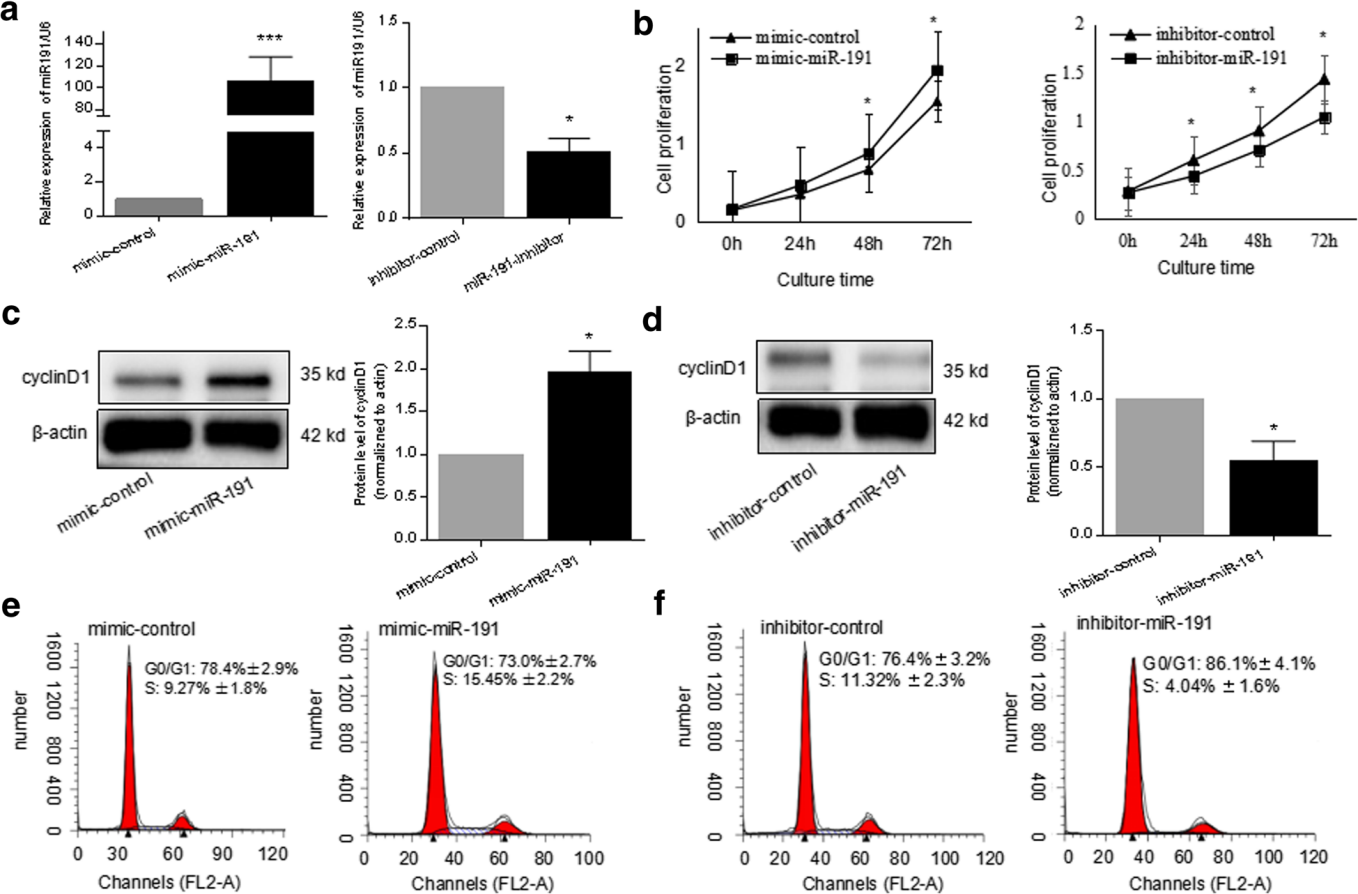
miR-191 promoted the cellular proliferation of RA-FLS. **a** Confirmation of the level of miR-191 in RA-FLS after transfected with mimic-miR-191 or inhibitor-miR-191 for 24 h by quantitative RT-PCR. **b** RA-FLS transfected with mimic-miR-191 and inhibitor miR-191 were plated at 4 × 10^3^ cells/well in 96-well plates; the cell viability of RA-FLS was determined by CCK8 at 0, 24, 48, and 72 h. **c** Increased expression of cyclinD1 in RA-FLS transfected with mimic-miR-191 for 48 h. Quantitative analysis on three repeats were presented. **d** Decreased expression of cyclinD1 at the protein level in RA-FLS transfected with inhibitor-miR-191 for 48 h. Quantitative analysis on three repeats was presented. **e** The cell cycle analysis by flow cytometry indicating the increased G_1_/S transition of RA-FLS by mimic-miR-191 transfection for 48 h. **f** The cell cycle analysis by flow cytometry indicating the decreased G_1_/S transition of RA-FLS by inhibitor-miR-191 after transfection for 48 h. All experiments were repeated three times. Data were presented as mean ± SEM (*n* = 3, ***p < 0.001, *p < 0.05 by *t* test)

MiR-191 was suggested to be an oncomiR by literature to be aberrantly expressed in various human cancers including breast cancer [20], hepaotocellular carcinoma [21], and colon cancer [22]. In order to elucidate the function of hypoxia-induced miR-191 in cellular proliferation in RA-FLS, mimic or inhibitor oligo of miR-191 was applied. As shown in Fig. 2a, transfection of mimic-miR-191 into RA-FLS increased the level of miR-191 around 100 times, whereas inhibitor-miR-191 decreased the miR-191 levels in RA-FLS efficiently. A CCK8 assay demonstrated the increased proliferation after mimic-miR-191 transfection. On the contrary, miR-191 inhibition decreased cell viability significantly (Fig. 2b). Western blot analysis demonstrated that the protein level of cyclinD1 increased after transfection with mimic-miR-191 (Fig. 2c) and significantly decreased in miR-191-downregulated RA-FLS (Fig. 2d). Cell cycle analysis indicated the promotion of G_1_/S transition in RA-FLS by miR-191 overexpression (Fig. 2e) and an attenuation of the G_1_/S transition by miR-191 inhibition (Fig. 2f).

### miR-191 inhibited starvation-induced apoptosis in RA-FLS

**Fig. 3:**
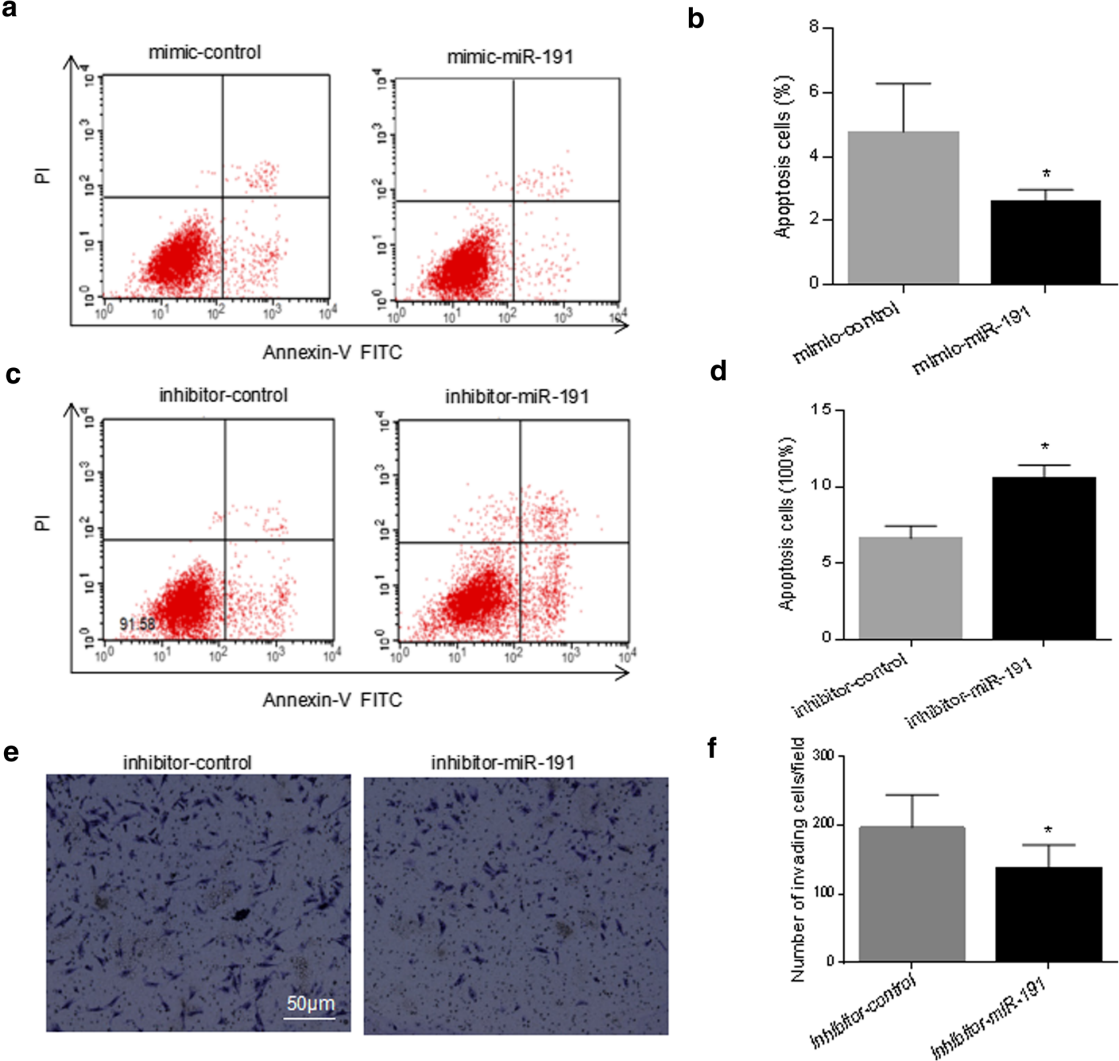
miR-191 inhibited apoptosis in RA-FLS. **a** RA-FLS were starved with 1% fetal serum for 24 h with or without overexpression of miR-191, followed by FACS analysis. **b** Bar graph showing the decreased apoptotic cell percentage after transfection with mimc-miR-191. **c** RA-FLS were starved with 1% fetal serum for 24 h with or without knockdown of miR-191, followed by FACS analysis. **d** Bar graph showing the increased apoptotic cell percentage after transfection with inhibitor-miR-191. **e** Transwell assays using RA-FLS cells after transfection with inhibitor-miR-191 for 24 h showed knockdown of miR-191 significantly suppressed the cell invasion of RA-FLS. **f** Quantitative analysis of e. All experiments were repeated three times. Data were presented as mean ± SEM (*n* = 3, *p < 0.05 by *t* test)

To determine the role of miR-191 in cell apoptosis, the Annexin V/PI analysis was applied to detect the apoptotic cells after transfection of mimic-miR-191 (Fig. 3a), followed by a quantitative analysis (Fig. 3b). Similar assays were performed after transfection of inhibitor-miR-191 (Fig. 3c, d). Quantitative analysis indicated significant decrease in the proportion of apoptotic cells from 6.37% to 3.45% by overexpression of miR-191 (Fig. 3b) and increase of apoptotic cells from 8.42% to 14.2% by knockdown of miR-191 (Fig. 3d).

Transwell assays were further applied to determine the cell migration/invasion regulation by miR-191 using RA-FLS cells with or without knockdown of miR-191. As shown in Fig. 3e, knockdown of miR-191 significantly suppressed the cell invasion ability of RA-FLS.

### C/EBPβ is a target gene of miR-191 in RA-FLS

**Fig. 4:**
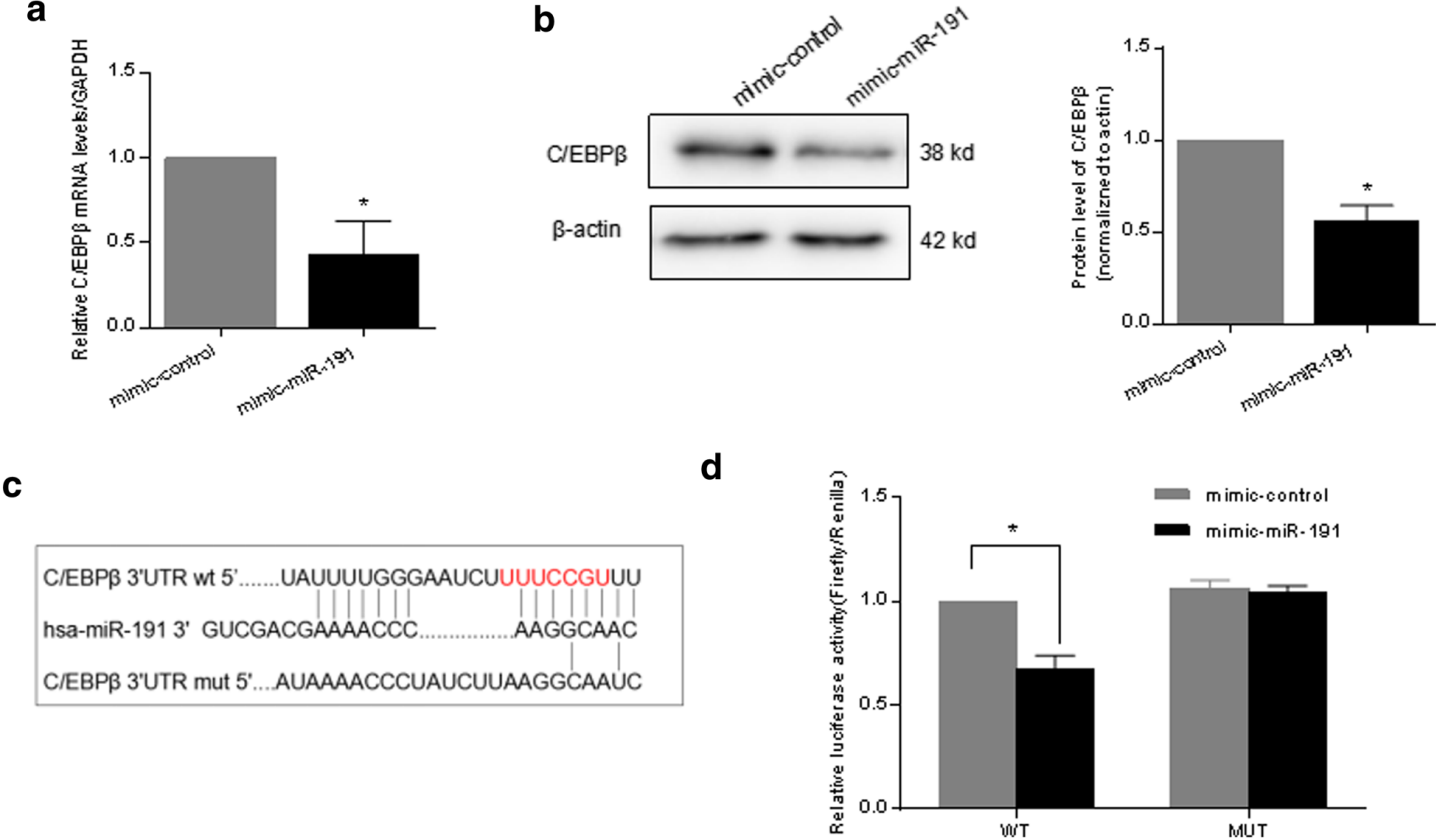
miR-191 regulates C/EBPβ expression in RA-FLS. **a** quantitative RT-PCR analyses demonstrated the decrease of mRNA levels of C/EBPβ in RA-FLS after transfected with miR-191-mimic for 24 h. **b** Western blot analyses demonstrated the decrease of C/EBPβ protein level in RA-FLS after transfected with miR-191-mimic for 48 h. **c** Sequences of the predicted miR-191 target sequences in the 3′-UTR of C/EBPβ and its mutant containing altered nucleotides in the 3′-UTR. **d** Luciferase reporter assays demonstrated the inhibition of C/EBPβ 3′-UTR by direct interaction with miR-191. All experiments were repeated three times. Data were presented as mean ± SEM (*n* = 3, *p < 0.05 by *t* test)

In order to determine the mechanism through which miR-191 regulate the proliferation of RA-FLS, we used three publicly available databases (TargetScan, picTar, and miRanda) to search for predicted direct target genes of miR-191. We found a binding site of miR-191 in the 3′-UTR of C/EBPβ mRNA. C/EBPβ is a member of the C/EBP family of transcription factors and has been reported to regulate cell proliferation, differentiation, and cell apoptosis in a variety of cells [23–25]. Quantitative RT-PCR and western blot demonstrated that overexpression of miR-191 could remarkably reduce the expression of C/EBPβ at both mRNA level (Fig. 4a) and protein level (Fig. 4b) in RA-FLS. The pMIR-REPORT luciferase vector was established using the wild type 3′-UTR of C/EBPβ and a point mutation sequence to the miR-191 binding site (Fig. 4c). As shown in Fig. 4d, miR-191 could directly suppress WT 3′-UTR of C/EBPβ, but not mutant vector.

### C/EBPβ mediated the miR-191 regulation of cell proliferation in RA-FLS

**Fig. 5:**
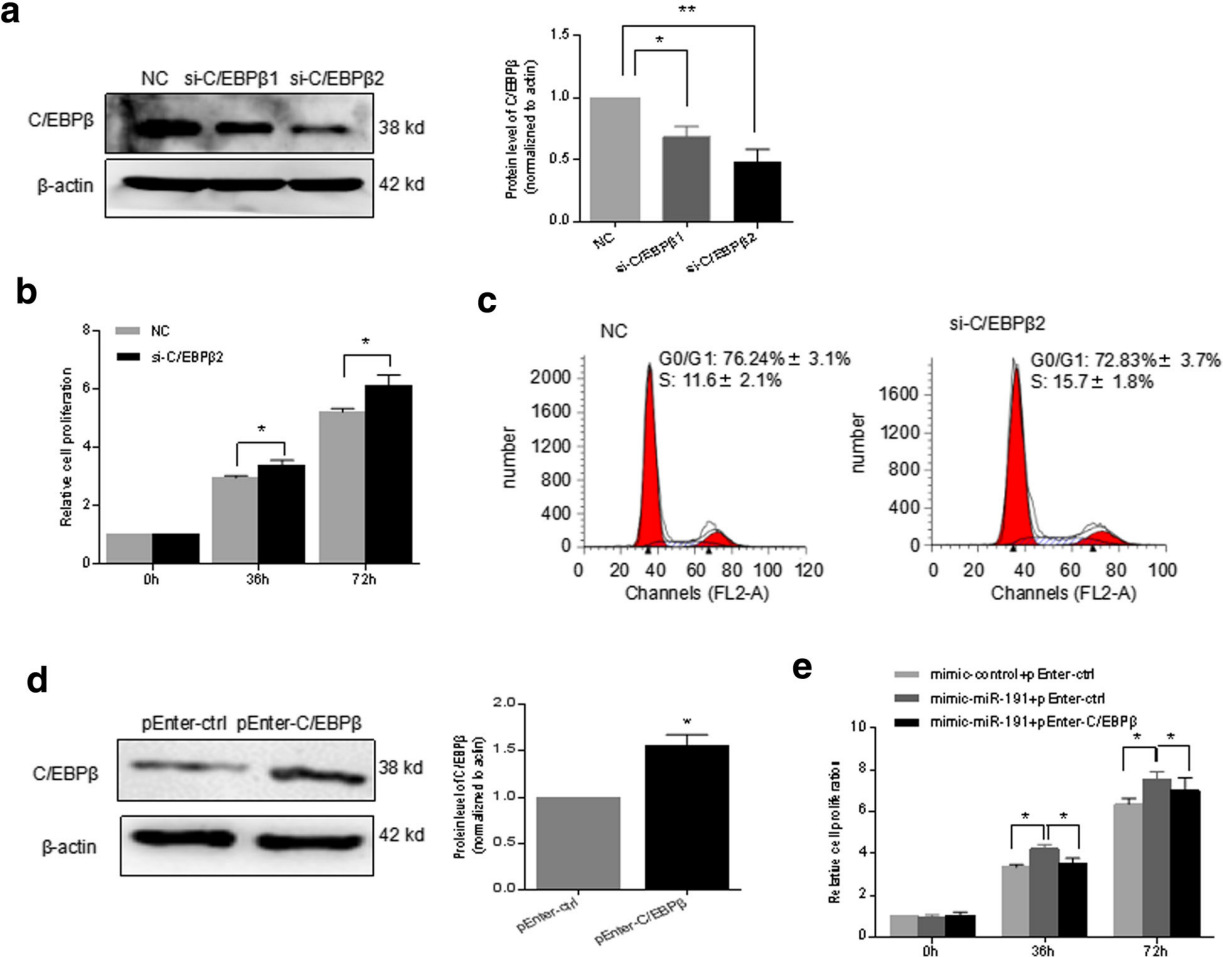
C/EBPβ mediated the miR-191 regulation of cell proliferation. **a** Western blot analyses demonstrated the knockdown of C/EBPβ in RA-FLS after transfection of siRNAs for 48 h. **b** RA-FLS transfected with NC or si-C/EBPβ2 were plated at 4 × 10^3^ cells/well in 96-well plates; the cell viability of RA-FLS was determined by CCK8 at 0, 36, and 72 h. **c** Cell cycle analyses showing the increased G_1_/S transition after knockdown of C/EBPβ for 48 h. **d** Western blot analysis of the levels of C/EBPβ in RA-FLS after being overexpressed with C/EBPβ for 48 h. **e** CCK8 assay showed that overexpression of C/EBPβ rescued the cell proliferation regulation by miR-191 in RA-FLS. All experiments were repeated three times. Data were presented as mean ± SEM (*n* = 3, **p < 0.01, *p < 0.05 by *t* test)

In order to determine the function of C/EBPβ as the target gene of miR-191 in RA-FLS, siRNAs targeting C/EBPβ (si-C/EBPβ-1 and si-C/EBPβ-2) were applied to knockdown the expression of C/EBPβ. C/EBPβ was knocked down by over 60% by si-C/EBPβ-2 as shown in Fig. 5a. Knocking down of C/EBPβ significantly promoted the cell viability (Fig. 5b) and increased the cell cycle G_1_/S transition in RA-FLS (Fig. 5c). pEnter-C/EBPβ, a vector overexpressing C/EBPβ (Fig. 5d), was applied to perform rescue assay. As shown in Fig. 5e, mimic-miR-191 increased the cell proliferation of RA-FLS, which was reversed by reintroduction back of C/EBPβ, indicating the regulation of cell proliferation by miR-191 in RA-FLS is dependent on the target gene C/EBPβ.

### C/EBPβ involvement in the regulation of hypoxia-induced cell proliferation in RA-FLS

**Fig. 6:**
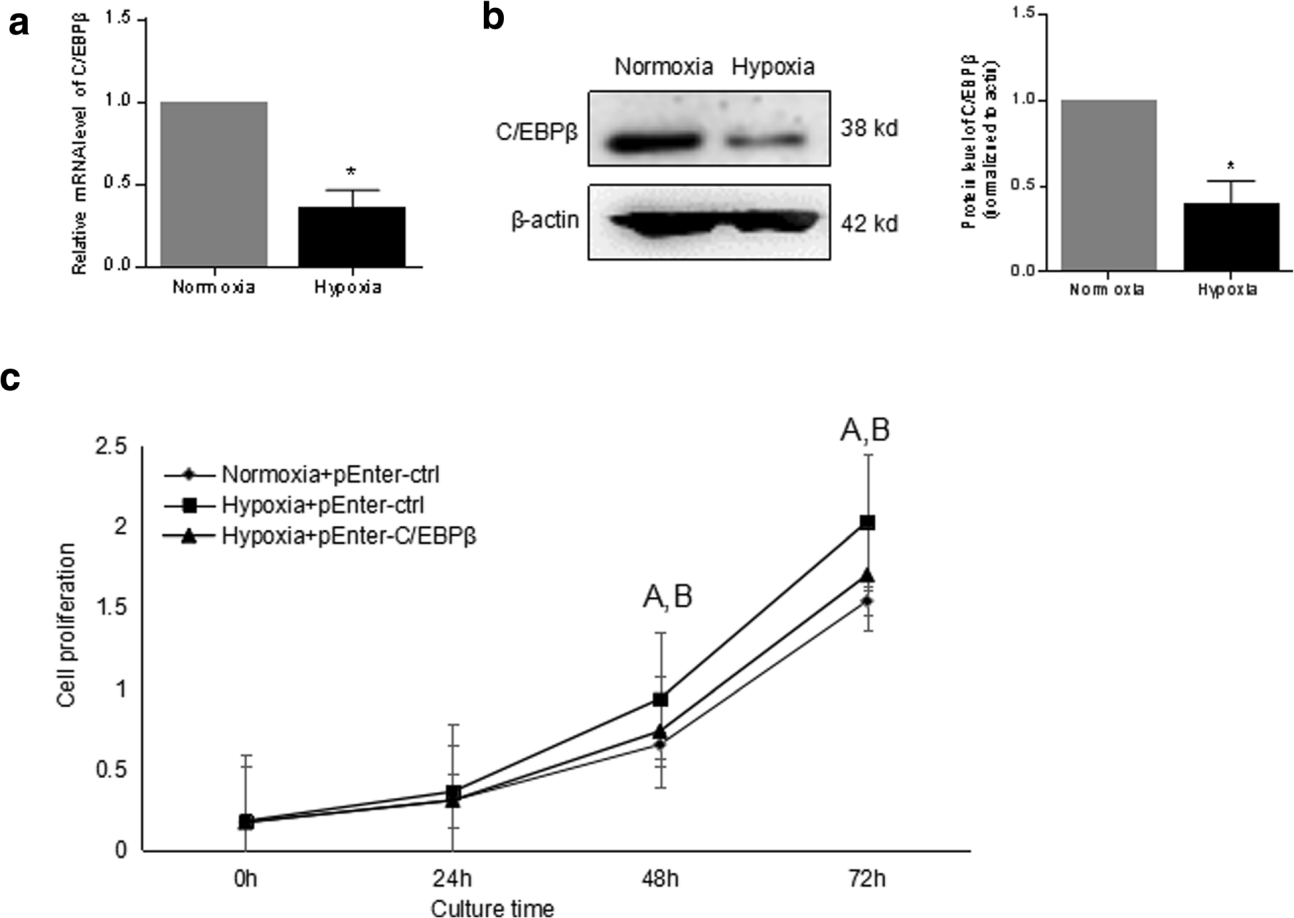
C/EBPβ involved in the regulation of hypoxia-induced cell proliferation. **a** quantitative RT-PCR demonstrated the decrease of the C/EBPβ mRNA level in RA-FLS cultured in 3% O_2_ hypoxia for 24 h. **b** Western blot demonstrated the downregulation of C/EBPβ at the protein level in RA-FLS cultured in 3% O_2_ hypoxia for 48 h. **c** Cell proliferation capacity was determined by CCK8 assay in RA-FLS overexpressed with C/EBPβ cultured under condition of normoxia or hypoxia. (A: Normoxia+pEnter-ctrl versus Hypoxia+pEnter-ctrl, *p* < 0.05; B: Hypoxia+pEnter-ctrl versus Hypoxia+pEnter-C/EBPβ, *p* < 0.05). All experiments were repeated three times. Data were presented as mean ± SEM (*n* = 3, *p < 0.05 by *t* test)

In order to further determine the role of C/EBPβ in RA-FLS, cells were cultured under hypoxia and normal condition, followed by gene expression assays. As shown in Fig. 6a and b, C/EBPβ was remarkably suppressed in expression by hypoxia at both mRNA and protein levels. Overexpression of C/EBPβ could partly rescue the hypoxia-induced cell proliferation (Fig. 6c). The findings above suggested that miR-191-C/EBPβ signaling is required to mediate the hypoxia-induced cell proliferation in RA-FLS.

## Discussion

Hypoxia has been well confirmed to be a common feature in most of the solid tumors [26]. It plays important roles in the development and progression of cancer [27]. Emerging evidence has demonstrated that it is inflammation and hypoxic microenvironment in the synovium that aggravates the cell proliferation and suppresses the cell apoptosis of synoviocytes in RA patients [28, 29].

In the current study, a miRNA screen analysis in the hypoxia-exposed RA-FLS was performed. We identified miR-297, miR-499b-3p, miR-770, miR-936, and miR-191 as the differently expressed miRNAs in RA-FLS under hypoxia condition. Interestingly, the upregulation of miR-191 was found to be specific in hypoxic RA-FLS, compared to hypoxic OA-FLS. The regulatory function of miR-191 to the cell proliferation and apoptosis of RA-FLS was further determined. We found miR-191-C/EBPβ mediated the upregulation of cell proliferation and inhibition of cell apoptosis in RA-FLS. Enforced overexpression of miR-191 was demonstrated to promote the proliferation and cell cycle and protect cells against apoptosis, suggesting miR-191 might be a potential target in RA treatment. In addition, upregulation of miR-191 was found in various types of cancer, autoimmune disease, and inflammatory disease [30–33]. In pancreatic cancer, miR-191 was found to suppress USP10, attenuated p53 stability, and thereby activate NF-κB signaling [34]. In consistence, proinflammatory cytokines including TNF-α, interleukin(IL)-1β, and IL-6, down-stream targets of NF-κB signaling, showed upregulation by miR-191 [34]. In view of the correlation between RA and inflammation, miR-191 is very likely to involve in the regulation of proinflammatory reaction in RA-FLS.

C/EBPβ has been shown to regulate cell proliferation, differentiation, and cell apoptosis in a variety of cell types [35, 36]. We found that C/EBPβ is a functional target of miR-191 in RA-FLS. MiR-191 targeted C/EBPβ regulating cell cycle inhibitors p16, p15, and p57 in colorectal cancer and thus is involved in the cell cycle regulation [22]. However, the miR-191-C/EBPβ interaction and the regulatory function in RA-FLS have not been reported yet. Herein we showed that C/EBPβ was downregulated by miR-191 as a target gene in RA-FLS, and knocking down of C/EBPβ promoted RA-FLS proliferation significantly. Reintroduction C/EBPβ back to RA-FLS after hypoxia treatment could rescue the hypoxia-miR-191-induced phenotypes, suggesting the cell proliferation and apoptosis regulation by hypoxia-miR-191 are mediated, at least partly, by C/EBPβ. However, rescue experiments did not entirely recover normal phenotypes, and further studies are needed to detect the role of other targets or factors in the hypoxia-induced cell function. In addition to C/EBPβ, ten-eleven translocation 1 (TET1) is also a predicted target of miR-191 in cholangiocarcinoma cells, which inhibits cell proliferation through p53 signaling pathway [37]. Whether miR-191-TET1-p53 signaling pathway is also involved in the hypoxia-regulated cell function in RA-FLS is going to be experimentally determined by our ongoing study.

Except for proliferation induction, hypoxia has been demonstrated to promote VEGF secretion from RA-FLS and regulate angiogenesis in RA [38]. miR-191 was also found to be involved in HIF-2α-induced angiogenesis [39]. In breast cancer, loss of C/EBPβ was reported to promote breast cancer progression by shifting TGFβ response [40], which is an extremely strong stimulator of VEGF production by synovial fibroblasts [41, 42]. Thus, these studies may be suggestive of the possible role of hypoxia-miR-191-C/EBPβ in regulating angiogenesis in RA, which needed to be further experimentally validated.

## Conclusions

Taken together, our findings demonstrate that hypoxia-induced miR-191-C/EBPβ signaling is required to mediate the cell proliferation and apoptosis of RA-FLS. These findings provide new insights into the role of hypoxamiR in RA and suggested miR-191 is a promising therapeutic target for RA.

## Abbreviations

3′-UTR: Three-prime untranslated region
ACR: American College of Rheumatology
C/EBPβ: CCAAT/enhancer binding protein β
CCK8: Cell Counting Kit-8
FBS: Fetal bovine serum
FLS: Fibroblast-like synoviocytes
G6PI: Glucose-6-phosphate isomerase
HE: Hematoxylin and eosin
HIFs: Hypoxia-inducible factors
hypoxamiR: Hypoxia-induced microRNAs
miRNA: MicroRNA
OA: Osteoarthritis
RA: Rheumatoid arthritis
RT-PCR: Real-time polymerase chain reaction
si-C/EBPβ: C/EBPβ siRNA
si-NC: Si-negative control
TET1: Ten-eleven translocation 1
VEGF: Vascular endothelial growth factor
